# Possible Causes of Hypertrophic Osteoarthropathy in the La Ferrassie 1 Neanderthal

**DOI:** 10.7759/cureus.35721

**Published:** 2023-03-03

**Authors:** Matthew D Turner

**Affiliations:** 1 Medicine, Madigan Army Medical Center, Lakewood, USA

**Keywords:** infectious disease, pulmonary malignancy, viral pneumonia, la ferrassie 1, neanderthal, hypertrophic pulmonary osteoarthropathy

## Abstract

For over a century, researchers have been perplexed by the unique osteological findings on La Ferrassie 1 (LF1), one of the most complete Neanderthal remains ever found. In 1997, Fennel and Trinkaus proposed that LF1 suffered from hypertrophic osteoarthropathy (HOA), likely secondary to chronic thoracic infection or pulmonary malignancy. This disease process can have many etiologies, and no study has fully explored the possible origin of LF1's HOA. Ultimately, it is most likely that LF1's HOA etiology arose from one of the many infectious diseases that prehistoric Neanderthals were exposed to, specifically a chronic pulmonary RNA virus.

## Introduction and background

From approximately half a million years ago to as recently as 30,000 years ago, *Homo sapiens*’ closest living hominid relative, *Homo neanderthalensis* (Neanderthals), lived in Europe and western Asia [1]. Neanderthals had a genome 99.5% identical to that of the Anatomically Modern Humans (AMH) [1] that they would interbreed with on at least two occasions [2]. They also displayed many complex behaviors similar to that of AMH, including the use of tools, burial of their dead [3], clothing [4], and even rudimentary healthcare [5].

One of the most famous Neanderthal remains is the La Ferrassie 1 (LF1) skeleton, discovered in 1909 at the La Ferrassie site in France. Over a century later, it remains one of the most complete Neanderthal skeletons ever found. The male Neanderthal, somewhere between 40 to 55 years old, was intentionally buried in a manner so well that nearly all of its skeletal structure was preserved, aside from the patellae and bones of the hands and feet. As with many other Neanderthal remains, LF1’s bones include a number of pathological lesions, from a mandibular abscess to possible osteoarthritic changes of the lumbar vertebrae [6]. Of particular note is Fennel and Trinkaus’s discovery in 1997 that LF1 exhibited bilateral periostitis of the distal femoral and tibial diaphysis, which they concluded was highly suggestive of hypertrophic pulmonary osteoarthropathy [7]. This disease process, now more commonly referred to as secondary hypertrophic osteoarthropathy (HOA), can develop due to a number of etiologies that include malignancy, chronic pneumonitis, pulmonary tuberculosis, and many more [8]. In this article, we strive to identify the underlying pathology that may have led to this skeletal manifestation in LF1.

## Review

Hypertrophic osteoarthropathy

HOA, involving the abnormal proliferation of the skin and osseous tissue of the extremities, typically manifests as a syndrome that includes digital clubbing, periostitis of the long bones, and joint effusions causing arthralgias through an inflammatory mechanism that is not fully understood. Primary HOA is an extremely rare hereditary condition, while secondary HOA (sometimes referred to as Marie-Bamberger syndrome) compromises the vast majority of cases. In the modern age, most causes of HOA can be attributed to paraneoplastic etiologies, with bronchial carcinomas alone accounting for 80% of HOA [9]. However, other disorders such as cirrhosis, pulmonary infections, sarcoidosis, cyanotic heart disease, inflammatory bowel disease, acquired immunodeficiency syndrome, and pulmonary tuberculosis [8] can play a role in the development of secondary HOA.

As Fennel and Trinkaus established, the bilateral periostitis present on the femurs and tibiae of LF1 is consistent with HOA [7]. Researchers have used similar radiological techniques to identify HOA-afflicted human remains from 1970 [9], medieval England [10], the Canary Islands roughly a millennium old [11], and Iron Age civilization [12]. While it is impossible to fully determine the diagnosis of secondary HOA, given that LF1 displays no soft tissue preservation, it appears highly likely that LF1 did experience this syndrome from an underlying pathology, theorized by Fennel and Trinkaus to be either a thoracic infection or a carcinoma [7]. In this paper, we compare the two possibilities to determine which is the more likely etiology for LF1.

An infectious etiology?

Based on the specimen’s extreme dental wear, Fennel and Trinkaus estimate LF1 to have been between the ages of 40 to 55 years, and thus elderly compared to other Neanderthal specimens. Given this, it appears possible that LF1 had “worn out their [body] and probably their resistance to infectious or degenerative processes” [7]. While it is impossible to determine the exact cause of LF1's extinction, it is likely that LF1's HOA, secondary to an underlying infectious or oncologic etiology, contributed to its death [7]. LF1's apparently deliberate burial, with no evidence of recent significant trauma [7], suggests that LF1 did not perish due to an inter-species rivalry for resources.

While the vast majority of modern cases of HOA in the modern age are due to an underlying malignancy [9], this appears to not have always been the case, particularly in the pre-antibiotic era. In 139 cases of secondary HOA published up to 1915, 76% were due to respiratory infection, with tuberculosis being an especially important cause of HOA [10]. It has been suggested that HOA has been severely underdiagnosed in the archaeological record and that paleopathologist, particularly when dealing with fragmented and disjointed remains, tend to “lump together instances of periosteal reaction in a skeletal series as non-specific skeletal infection, without adequate consideration of other possible causes such as HPO [HOA]” [10]. Given LF1’s advanced age, it is possible that its HOA could have been due to an acute infection taking advantage of its weakened immune system. This conclusion is further supported by the absence of metacarpal, metatarsal, or phalangeal periostitis, which suggests that LF1’s HOA had not yet progressed to the more advanced stages. Fennel and Trinkaus propose that LF1 had suffered from HOA that began only 2-14 months before death [7], a timeline that is consistent with an acute thoracic infection.

Neanderthal healthcare

In order to properly diagnose LF1, an overview of Neanderthal healthcare is required. Over 200 Neanderthal remains have been identified [13], a remarkable number since conservative estimates of their effective population size range from 1,000 to 3,500 [14]. Of these remains, almost every specimen that survived past the age of 25 to 30 years have at least some evidence of a post-traumatic lesion, with an exceptionally high frequency of head and neck injuries. This is particularly pronounced in males, implying a sexual division of labor for the dangerous task of hunting. One study estimated that Neanderthals had a frequency and pattern of trauma similar to that of professional rodeo athletes [15]. However, AMH of the same era exhibited a similar prevalence of cranial trauma, suggesting that the hunter-gatherer lifestyle of the era was equally dangerous for both species [16].

One notable example of this is the famous St Césaire Neanderthal. In this male specimen dating to approximately 36,000 years ago, there is evidence of a healed fracture in the cranial vault. This fracture occurred due to a direct, sharp blow with a blade-shaped implement, likely caused by interpersonal violence [17]. However, just as importantly, the bone regeneration evident in the specimen indicates that the Neanderthal survived - a grueling process that may have taken weeks or even months, during which he would have required significant care from the others in his group [5]. One of LF1’s injuries - a healed fracture of the right femoral greater trochanter [6] - would likely have required a similar degree of care, during which the specimen would have had significantly reduced immobility. LF1’s left clavicle is also concerning for a possible well-healed fracture, possibly a greenstick fracture sustained in childhood [6] that would have also required significant care. While AMH males who suffered similar cranial traumas were more likely to survive into old age than equivalent Neanderthals [16], the evidence available to us does suggest that the Neanderthals routinely practiced healthcare as “part of a social context of strong pro-social bonds” in a “compassionate and knowledgeable response to injury and illness” [5].

Care for the wounded, elderly, or infirm in the group predates both Neanderthals and AMH. An *H. erectus *individual from 1.7 million years ago suffered from a such significant periodontal disease that they were completely edentulous, yet still survived a significant length of time, likely due to the aid of others in their group [18]. Healthcare behaviors can be traced even earlier - *H. habilis *from 2.4-1.6 million years before the present day often have evidence of deep grooves in their teeth, and wood fragments present in their dental calculus, possibly from the use of makeshift toothpicks to treat periodontal disease [18]. Many Neanderthal teeth express the same pattern of interproximal grooves, indicating dental probing [5,11]. In addition to this, the Neanderthals may have even used medicinal plants. A 49,000-year-old individual from Spain suffering from a dental abscess was found to have evidence of two medicinal plants of minimal nutritional value in their dental calculus - of note, there was evidence of chamazulene, a biochemical associated with a bactericidal and anti-inflammatory effect [18]. A separate study on the same individual found sequences corresponding to poplar, which contains salicylic acid, the active ingredient in aspirin [19]. Far from being carnivores, the Neanderthals had a significant plant intake in their diets as well, as evidenced by the fecal biomarkers they left behind [20]. In a similar way to how chimpanzees eat strong, bitter leaves when consuming the chyme of their prey [21], Neanderthals may have instinctively used plant life to “flavor” their food and reduce the risk of infection [22].

However, the evidence currently available to us suggests that Neanderthal healthcare was far from instinctual in nature - it was likely a deliberate system of complex social interactions that evolved over time. In an analysis of Neanderthal archaeology, researchers concluded that the species developed an increasing number of and diversity of complex behaviors - including evidence of heavily modified raw materials, the burial of dead, multi-component tools, decorative pigments, and even the body modification examples of Shanidar 1 and 5 mentioned above - throughout their history, with an exponential increase in complex behaviors between 60,000 to 40,000 years ago. Though this could be due to simple differential preservation (with greater preservation of artifacts from 40,000 years ago than those from 140,000 years ago) and changing population densities, the researchers concluded that Neanderthal behavior was undergoing "changes toward greater economic and technological flexibility and complexity, involving increasing rates of innovation and diversification” [3]. While this specific paper does not delve into the specifics of Neanderthal healthcare, a steadily increasing level of social, economic, and technological progress implies an increasing complexity of healthcare as well.

Neanderthal healthcare seems to have peaked at a level of care familiar to the modern world, with long-term care, such as for the St Césaire individual, lasting weeks to months, covering everything from providing food to “fever management, hygiene maintenance, repositioning, and manipulation.” The most intensive cases would have used up significant resources and would have not necessarily been to the benefit of the larger group [5]. Even so, the Neanderthals “had a medical competence, a finding consistent with a pattern of high rates of healing and low levels of infection” [5].

There is evidence to suggest that Neanderthals even practiced artificial cranial deformation. Shanidar 1 and Shanidar 5, 2 specimens from Shanidar Cave in Iraq that date to 45,000 years ago, display an interesting pattern of deformation in their cranial vaults - both displaying a flat frontal arc and a significantly curved sagittal arc at the very limits of known ranges of variation. Trinkaus suggests that this unusual combination was highly unlikely to have formed through either natural phenotypic variation, significant trauma, or post-mortem distortion [23]. It is possible that this unusual skull morphology may have been produced by the same processes of artificial cranial deformation that have been seen throughout *Homo sapiens*' history, from the elites of pre-Columbian America, the Merovingians of ancient Gaul, to the upper class of nineteenth-century France and Switzerland, in a tradition of body modification which has been "in existence since the origin of mankind" [24]. In the specific case of these two individuals, Trinkaus suggests the use of bands [23]. Perhaps the Neanderthals shaped the heads of their infants in a manner comparable to the cloth bands used in the 19th century Seine region of France, or perhaps they used head pressing techniques planks closer to the Chinook and Cowlitz Native Americans of the Pacific Northwest [24].

There appear to have been limits to the efficacy of Neanderthal healthcare. There are examples of Neanderthals that survived amputation, such as the unfortunate “Nandy”, a male who lived for years after a partial amputation of one of his arms. He compensated for the loss of his limb by using his worn-down mandibles to grip things afterward, but in this case and all the others known to us, these amputations seem to have universally been the results of accidents or trauma, not a deliberate form of crude surgery [25]. Similarly, although some researchers have theorized that the AMH of the time could have been aware of sutures, given their use of sewing and bone needles, no needles have ever been found at Neanderthal sites. Sewing itself may have been a mystery to them - it appears that their clothing, likely formed by tying together strips of skin [26], was cape-like, as opposed to the more form-fitting garb used by the AMH of the day [4].

Infectious diseases in Neanderthals

While skeletal evidence for trauma in Neanderthals is remarkably prevalent [15], it is significantly more difficult to preserve evidence of infectious diseases in bone. One exception can be found in a 60-year-old specimen, who suffered from a pattern of erosive defects and reactive new bone formation in his lumbar vertebrae, a pattern that is consistent with Brucellosis. *Brucella *was likely widespread in the animal populations of the time, and it is possible that Neanderthals were exposed to this pathogen either through butchering or eating raw meat. If correct, this could be the earliest known example of this zoonotic disease [27]. Exposure to zoonotic diseases was likely common among the Neanderthals due to both their lifestyle and diet. As a result, the species had a protective mutation in the form of a deletion in their bone marrow stromal antigen 2 (BST2) antiviral factor, one that likely provided them a major immunity against simian immunodeficiency viruses (SIVs) such as the direct ancestors of modern HIV [28]. Of note, AIDS has been identified as a possible cause of HOA in the modern era [8] - could one of the SIVs of the distant past, to which their immunity implies that Neanderthals were likely exposed, have caused LF1’s syndrome?

Evidence of other pathogens has lingered in the remains that have survived to the present day. The Neanderthal from Spain with the dental abscess discussed earlier, also tested positive for *Enterocytozoon bieneusi,* a chronic gastrointestinal pathogen that would have likely caused significant diarrhea [19].

Further examination of Neanderthal anatomy reveals hints of other infectious etiologies that may have afflicted the hominids. In a 2019 paper, researchers concluded that the abnormal morphology of the Neanderthal Eustachian tube may have predisposed them to high rates of otitis media. The Eustachian tubes of adult Neanderthals were closer in structure to the Eustachian tubes of AMH infants, possibly leading to a lifelong vulnerability to middle ear infections. Nearly 100% of Neanderthal specimens today do indeed display the ossicular pathologies that would be expected from chronic otitis media [29].

The Neanderthals may have also been afflicted by malaria. The disease emerged over 100,000 years ago before the *H. sapiens*' dispersal out of Africa [30], and it is possible that encroaching AMH may have exposed Neanderthals to it. Both AMH and Neanderthal populations of the Middle Paleolithic era predominately stayed in arid environments that would have offered a degree of protection against malaria - it may be that the disease affected the distribution of settlements just as much as the changing climate did [31]. Some researchers have even theorized that the distinct European strain of Plasmodium vivax could have persisted for millennia as a parasite specific to Neanderthals before it jumped to AMH [32].

Researchers have proposed a number of other pathogens that may have afflicted Neanderthals, including *Helicobacter pylori*, mycobacterial infections, pertussis, pinworms, tapeworms, adenoviruses, flaviviruses, Hepatitis A, HPV, rabies, and several others [14]. One of the most intriguing possibilities is that raised by Underdown, who proposed that Transmissible Spongiform Encephalopathies (TES) may have played a significant role in the hominid’s extinction [33]. At a number of sites, Neanderthal remains have been found with modifications that include “scraping, percussion marks, fragmentation, and burning… the same patterns of modification as processed game animals” [13]. While many researchers contend that that is due to one of the hominid’s well-documented mortuary practices, in many cases the remains are consistent with “defleshing and dismemberment” [13]. In one study of 9 sites across Europe that exhibited this pattern, researchers found that the remains were often of “distinct social units” compromising adults, children, and infants - all violently slain in a pattern of mass killings and subsequent modification of the dead that was strongly suggestive of “cannibalism, either nutritional or ritual” [13]. The processing of the dead was often done in direct parallel to the processing of other prey animals. At the cave site of Moula-Guercy, researchers found a similar pattern of defleshing and disarticulation between three Neanderthal remains and the surrounding deer bones. Interestingly, the researchers concluded that even after the incident of cannibalism, the cave continued to be occupied - nor were the Neanderthal bones altered in any way that would suggest some sort of ritualistic process [34]. Whatever the reason, it appears that cannibalism was relatively widespread amongst the Neanderthal population, which Underdown suggests would have put them at risk of a number of TES [33].

RNA viruses

Much of the greatest evidence for Neanderthal’s diseases can be found in the genome of *H. sapiens*. AMH and Neanderthals interbred on at least two occasions [2], and Neanderthal-derived genes make up 0.5%-2% of the genome of most living humans, with a higher percentage in non-African populations [35]. Much of the Neanderthal genome was quickly removed from the *H. sapiens* genome through the process of purifying selection, rapidly dropping the proportion of Neanderthal genes from approximately 10% to their modern levels [2]. This is likely due to the small gene pool of the Neanderthal population, which would have likely led to significant inbreeding and reduced fitness [36], and thus less opportunity to purge deleterious mutations from their genome [37].

While the vast majority of Neanderthal-derived genomes were purged from the AMH population, a number of their sequences were introgressed into the modern genome. One study found that Neanderthal-derived genetic sequences are highly enriched in virus-interacting proteins (VIPs), which form a vital component of the immune response. Neanderthal VIPs that interact with RNA viruses are particularly favored to have been retained in AMH populations. Through the “poison-antidote” model, researchers hypothesized that, as gene flow occurred between AMH and Neanderthals, they also exposed one another to new pathogens that the exchanged sequences offered protection against. The strong enrichment of Neanderthal VIPs suggests that RNA viruses acted as a particularly powerful form of natural selection on the hominids [2]. One specific example of this is the oligoadenylate synthetase (OAS) locus on chromosome 12, which plays a crucial role in the innate immune defense against viruses, specifically those of the Flaviviridae family. This viral family includes West Nile virus, Hepatitis C, and tick-borne encephalitis [35], suggesting that Neanderthals may have been exposed to the direct ancestors of these modern diseases. This same OAS haplotype also offers protection against becoming severely ill with COVID-19 [38,39], further suggesting that Neanderthals were exposed to frequent and powerful RNA virus epidemics.

While other Neanderthal sequences were also introgressed into the genome of AMH, including alleles that enhanced the expression of subcutaneous adipose tissue [37] and skin pigmentation [40], VIPs associated with RNA viruses had an exceptionally strong selective pressure, far more than VIPs associated with DNA viruses [41]. This is perhaps explained by the nature of RNA viruses - they are highly adept at jumping from one species to another, and the vast majority of zoonotic diseases in human history have been RNA viruses [41]. Although we know little about ancient viruses, especially since RNA degrades much more quickly than DNA [41], it is evident that RNA viruses have likely existed in vertebrates for hundreds of millions of years. One study found 214 RNA viruses in a spectrum of vertebrate samples that ranged from reptiles to amphibians to jawless fish [42]. Given their low population densities [14] and largely meat-based diet [19], it is highly probable that the vast majority of infectious diseases Neanderthals experienced were zoonotic in nature, an environment that would have favored RNA viruses over other infectious agents.

Unfortunately, the evidence available to us is still lacking on the specific RNA viruses that Neanderthals may have encountered. RNA viruses have a genome on average that is only 15,000 nucleotides long and have a mutation rate that is typically 1,000,000 times faster than that of vertebrates [43]. This allows for exceptionally swift adaptations and the development of new pathogens but means that, in conjunction with the accelerated degradation of RNA [41], it is extremely difficult to determine more detailed information about the viral pathogens that afflicted the prehistoric world.

However, with the recent sequencing of the Neanderthal genome, this may soon change. Human endogenous retroviruses (HERVs) are the remnants of ancient RNA viral infections of germ cells that were subsequently incorporated into their host’s DNA. Long regarded as “junk” DNA, they are now being used to gather evidence about ancient infections [44]. In 2012, researchers analyzing the Neanderthal and Denisovan (another archaic hominid) genomes found evidence of 3 sequences where Human Endogenous Retrovirus K (HERV-K) had integrated into the Neanderthal genome [45]. While these sequences were also found to be present in the *H. sapiens* genome by another team of researchers [46], another study two years later found two HERV-K provirus elements unique to Neanderthals, and three HERV-K provirus elements found in both Neanderthals and Denisovans but not in the *H. sapiens* genome [47]. In modern *H. sapiens*, HERV-K elements have been suggested to contribute to a range of pathologies, including ovarian cancer, prostate cancer, melanoma, and systemic lupus erythematosus. Interestingly, no current infectious counterpart to HERVs has been identified in the modern day [44]. There are two possibilities from this: either the difference in HERV-K elements between humans and Neanderthals is due solely to genetic drift, or the RNA virus responsible for HERV-K was still replicating and integrating into the Neanderthal genome before going extinct relatively recently [48]. Until further data are found, the answer is likely to elude researchers.

Of all the various infectious diseases that likely afflicted Neanderthals, the one most likely responsible for LF1’s HOA was likely a zoonotic RNA virus. While viral pneumonia is relatively rare during early adulthood, LF1’s advanced age would have put him at greater risk of a ral pneumonia, perhaps comparable to the pneumonia caused by influenza and respiratory syncytial viruses in senior populations today [49]. While Neanderthals were exposed to other pathogens, their low population densities, high level of engagement with wildlife, and genetic lineage [41] all suggest that RNA viruses were a significant source of morbidity and mortality for them. Given this, it is possible that LF1’s HOA may have been secondary to a chronic viral pneumonia.

An oncologic etiology?

Aside from a thoracic infection, the other most likely cause that Fennel and Trinkaus identified for LF1’s HOA was a possible pulmonary malignancy. For decades, researchers maintained that oncogenic tumors, if they were ever present at all, had a minimal presence on the health of ancient hominids, due to the lack of obvious carcinogenic risk factors and the relatively young age of these populations. Approximately 60% of hunter-gatherers of the 21st century do not survive to reproductive age [50]. Although this is not a perfect analog for Neanderthal populations, it is difficult to imagine that oncogenic tumors were a significant threat to their health compared to the trauma [15] and infectious disease [41] that they routinely faced. As of 2010, only a single example of a Neanderthal neoplasm had been identified - a possible meningioma present on a skull fragment from Stetten, Germany [51].

However, recent discoveries have thrown this assumption into question. In 2013, researchers identified a fibrous dysplasia on a rib from another Neanderthal dating to 130,000 years ago. Another Neanderthal from 35,000 years ago has been identified with ossifying sarcoma, in addition to a number of other ancient hominids that have been diagnosed with various forms of osteogenic tumors [50]. Researchers have theorized Neanderthals were exposed to *Helicobacter pylori*, HPV, and human herpesvirus 8 [14], all of which have oncogenic properties [50]. Interestingly, the HERV-K provirus has been tied to various cancer etiologies in modern humans as well [44], including ovarian cancer, melanoma, breast cancer, prostate cancer, lymphomas, leukemia, and sarcomas [52]. As discussed above, whether through recent infection or genetic drift, HERV-K had a slightly different manifestation in Neanderthals as well [47] and may have acted as an oncogenic factor within the hominid.

Another important factor to consider is the relative age of LF1. As an elderly male [7], LF1 would likely have been at a higher risk of developing a neoplasm than other Neanderthals. The possibility that LF1 experienced some form of intrathoracic malignancy responsible for its HOA is thus greater than it would initially seem.

The final determination

From the evidence available to us, it appears that Neanderthals experienced both infectious and oncogenic pathologies that would have been capable of inducing HOA. The final piece of evidence from LF1 is a bony fragment that was not covered in Fennel’s and Trinkaus’ initial analysis: the pathological anomaly noted in a 2018 study on LF1’s sixth or seventh left rib. It presents as two abnormal bulges on the internal surface of the rib, while the exterior surface of the rib displays an area of bone remodeling. The researchers suggested that this was either due to previous trauma or to LF1’s HOA [6]. While a healed injury is possible, given the Neanderthal propensity for trauma [15], it is unlikely that this lesion is due to LF1’s HOA. None of the other ribs display this pattern of bone growth and remodeling, implying the pathology was focal, not systemic in nature.

The two “abnormal bulges” noted on the internal surface of LF1’s rib [6] resemble similar lesions found on the visceral surface of a medieval skeleton that also displayed HOA. In the medieval case, researchers concluded that the lesions, partially remodeled deposits of new bone, arose “from contact dissemination from underlying pleuro-pulmonary infective foci” that was ultimately responsible for the specimen’s HOA [10]. A similar case, also featuring new visceral rib growth in the setting of HOA due to a chronic pulmonary disease, has been identified from 2nd - 3rd century Sudan [53]. Chronic pulmonary infections such as tuberculosis have been shown to cause rib lesions [10,54].

While LF1’s left rib does display remodeling on its exterior aspect in addition to the two abnormal bulges on its visceral aspect [6], it does not display the concentric thickening seen in HOA-affected ribs, nor do any other ribs resemble this pathology [10]. We suggest that this rib pathology is emblematic of an underlying focal area of pneumonia, possibly the one responsible for the development of LF1’s HOA. Given this, and the infectious etiology of most cases of HOA in the pre-antibiotic age [10], it appears likely that LF1’s HOA was not due to malignancy but due to a chronic pulmonary condition. For the last 2-14 months of LF1’s life [7], the Neanderthal may have suffered from chronic pneumonia, possibly viral and zoonotic in nature, that triggered a systemic inflammatory periostitis in LF1’s long bones.

## Conclusions

Although the vast gulf of time makes it impossible to conclusively determine the true etiology of LF1’s HOA, the evidence available to us implies that the Neanderthal’s condition was an inflammatory reaction due to a chronic pulmonary infection. Already weakened by old age and pneumonia, the pain LF1 must have experienced from the periostitis in his legs may have been more than the Neanderthal healthcare system could handle. Whether due to pneumonia infecting his lungs or some other factor, LF1’s death before the HOA could fully advance may have not been a coincidence. Through further study of the diseases that afflicted other hominids, we may grow to better understand the nature of *H. sapiens *as well.
